# The Effect of Organic Mulching and Irrigation on the Weed Species Composition and the Soil Weed Seed Bank of Tomato

**DOI:** 10.3390/plants9010066

**Published:** 2020-01-03

**Authors:** Renáta Petrikovszki, Mihály Zalai, Franciska Tóthné Bogdányi, Ferenc Tóth

**Affiliations:** 1 Faculty of Agricultural and Environmental Sciences, Plant Protection Institute, Szent István University, H-2100, Páter Károly u. 1., H-2100 Gödöllő, Hungary; petrencsi@gmail.com (R.P.); Zalai.Mihaly@mkk.szie.hu (M.Z.); 2 FKF Nonprofit Zrt. H-1081, Alföldi u. 7., H-1081 Budapest, Hungary; T.Bogdanyi.Franciska@gmail.com

**Keywords:** integrated weed management, leaf litter mulch, weed ecology, germination tests, weed seed bank

## Abstract

Mulching is a management technique to control weeds in organic and integrated tomato production. Our experiment was designed to investigate the impact of organic mulch combined with irrigation on the weed species composition and weed seed bank of open-field tomato. For three consecutive years (2016–2018), treatment of microplots included mulch only, irrigation only, mulch and irrigation combined, and the untreated control. Marginal microplots (bordered by the surrounding mown grassland) were distinguished from inner microplots to check margin effect. We collected soil samples from different depths and let the weed seeds germinate in a greenhouse. Germinated weed seedlings were counted and identified. The number of weeds, and time needed for weeding was reduced by mulching, temperature, sampling date, and the succession of the study years. Irrigation, on the other hand, had no effect on weeding time. Margin effect and year had the highest influence on weed species composition. Regarding seed bank, year and mulching had the highest influence. The importance of other variables remained low, with mulching being the strongest explained variable. Regardless of treatments, weed composition of the study area was transformed during the three-year study.

## 1. Introduction

Weeds compete with crops for space, light, nutrients and water, and may host pathogen agents of cultivated crops. In sustainable and organic farming, weeds and the lack of conventional weed control measures mean considerable obstacles to production [1,2].

Weed species composition of organic tomato (*Lycopersicon esculentum* Mill. L.) production in an open-field environment is influenced by the location of the field, the characteristics of climate and soil, and by management techniques as well [3,4]. The list of the most common weeds of open-field tomato in Hungary includes *Ambrosia artemisiifolia* L., *Setaria* spp. Beauv., *Amaranthus* spp. L., *Chenopodium album* L., *Echinochloa crus-galli* (L.) Beauv., *Polygonum aviculare* L., *Portulaca oleracea* L., and *Solanum nigrum* L. [5].

Appropriate weed management requires the knowledge of the weed seed supplies of a field [6]. One of the most important sources of weeds is the seed bank of the soil: a depot of seeds and in a broader sense, of other plant propagules. Seeds in the seed bank may either be freshly shed or have remained within the soil for years [7]. Seed bank is where seeds are deposited and protected from unfavorable environmental conditions over an extended period and maintain their viability for years or even decades [6].

Seed size is an influential factor of germination: the viability of bigger seeds is higher in the deeper layers. Moreover, mortality of monocotyledonous species is higher than dicotyledonous species in the same depth [8]. This is because larger seeds have more seed mass than small ones [9]. Smaller seeds are generally more sensitive to allelochemicals [10] and smaller seeds of the same species are more sensitive to environmental changes [11].

The persistence of seeds, and thus the successful reproduction of weeds in the field is influenced by several environmental factors [12] including soil temperature, pH, moisture, and light [13]. Shading by crops modifies temperature and light. Different weed species react differently: *Galinsoga parviflora* Cav. germinates easier in comparison to *Amaranthus quitensis* Kunth and *P. oleracea* [14]. The success of mulch materials in weed control is partly attributed to their ability to block the sunlight from reaching the soil surface. The germination and further development of weeds that need light for their germination or early development are therefore hindered or completely halted [15]. The detection of favorable conditions for germination is a key factor to have an effective weed control [12] but in organic weed control, knowing the weed seed bank of an area is also important [1]. Surveying the weed seed bank of the soil may start with extracting seeds directly from the soil or germinating weed seeds from soil samples [16]. The larger the number of samples, the more representative the germination test of the actual seed bank composition [6].

There are direct and indirect approaches in sustainable weed control. Mechanical weed control, thermal weed control, mulching, and biological weed control methods are means of direct weed control, whereas indirect methods include, but are not limited to the selection of crop cultivars that tolerate or suppress weeds, the use of intercropping or mulch materials, crop rotation, and cultivation [1,17]. Management techniques such as tillage or lack of tillage combined with mulching influence weeds and their viability. In case of certain species (*Amaranthus* spp., *Cuscuta* spp. L.), seed viability was lower in soil samples under mulched areas than in tilled plots [18]. For example, the germination of *Amaranthus retroflexus* L. seeds was slower under rye mulch than in control plots. However, mulching with poplar (*Populus deltoides* Bartr.) did not influence *E. crus-galli*, since poplar mulch is chemically inert, no phytotoxic compounds leach from it [19]. Rye (*Secale cereale* L.) straw, on the other hand, may release allelochemicals or phytotoxic acids that decrease the germination of weed seeds [10,20,21]. Rice straw mulch was almost as much effective as polyethylene [22]. Straws of other origins such as rye, or buckwheat (*Fagopyrum esculentum* Moench.) were proven to suppress weeds [23,24] due to their allelopathic effects not only on seed germination but on growth as well [20]. Besides straw, peat and sawdust can also reduce the presence of perennial weeds, but other mulch materials, such as grass clippings for example decompose faster, leading to a decline in their efficacy in the second year [25]. A study investigating the effect of mulching in weed control [26] found that compost, weed and alfalfa clippings are not as efficient as paper, black plastic covering, or rye straw. Depending on the origin of the composted materials, acetic acid and organic acids may leach from compost. These compounds may be phytotoxic to weeds and certain crops as well [27].

### Objectives

Leaf litter of deciduous trees is a major constituent of municipal green waste in Hungary, collected in large quantities every autumn by waste management companies, but, according to our recent unpublished survey, no effective agricultural or horticultural uses have been found for this material. Considering leaf litter as a massive, valuable and yearly generated resource, we decided to investigate its potential in weed control.

The present study was planned to determine the effect of organic mulching combined with drip irrigation on the weed species composition and weeding time requirement of tomato microplots in open-field conditions. Our additional aim was to survey the weed seed bank of the soil by collecting samples from different depths of soil and germinating weed seeds under greenhouse conditions.

## 2. Results

### 2.1. Open-Field Experiment

All examined parameters (mulching, margin, seasonality, year, rainfall, temperature, and irrigation) had significant influences on weed composition. Year and margin effect accounted for the highest variance. Both mulching and margin had noticeable gross and net effects. The highest difference between gross and net effect was observed in the case of sampling date (time of season) and temperature (Table 1).

Mulching significantly decreased the emergence of most weeds. Among species that fit the pRDA model the best, only the occurrence of *Convolvulus arvensis* L. increased. (Table 2) While most species were dominant at the beginning of the season, *Digitaria sanguinalis* (L.) Scop. and *Glechoma hederacea* L. were more frequent at the end of the growing season. *Elymus repens* (L.) Gould was frequent during the first surveys, but the species disappeared later (Table 2).

We found that temperature before the surveys influenced weed composition: while *P. oleracea*, *C. arvensis* and *D. sanguinalis* were dominant in warm periods, *Stellaria media* (L.) Vill. preferred colder weather (Table 2).

Weeds that prefer undisturbed habitats, for example *G. hederacea*, seemed to favor microplots near margins, and species with shallow-located rhizomes such as *E. repens* and *Cynodon dactylon* (L.) Pers. appeared on inner and marginal microplots as well. Regardless of the presence of mulch, rainy weather encouraged *E. crus-galli*, *P. oleracea* and *D. sanguinalis*, while certain weeds, *E. repens* and *Setaria* spp., appeared only in the low rainfall period. There were changes to the weed composition of the study area between the consecutive years too. While certain typical annual arable weed species, such as *S. media*, *D. sanguinalis*, *Ch. album*, *G. parviflora*, and *Setaria* spp., were dominant in the first year (2016), the perennial *G. hederacea* became frequent from the second year (2017) on, and perennials *E. repens* and *C. dactylon* became frequent only in the third year (2018) (Table 2).

Weeding time was not influenced by margin, temperature or irrigation, but mulching decreased the time required for weeding. In addition, less time was needed for weeding as the growing season progressed (Table 3).

Total weed coverage changed and was significantly decreased by mulching, the succession of study years, temperature, and sampling date (Table 4).

The ordination diagram of RDA of our open-field experiment indicates a high correlation between temperature and sampling date (time of season), but the effect of margins seemed to have no correlation with these variables (temperature and sampling date). The effect of mulching was similar, only less significant than the effect of margin on weed composition. The first year (2016) is characterized by a higher abundance of summer annuals (*Ch. album*, *G. parviflora*, *Setaria viridis* (L.) Beauv.). *Solanum tuberosum* L., the preceding crop, and *Acer platanoides* L. seedlings were also recorded in large numbers. The years 2017 and 2018 were more similar to each other in both being more affected by sampling date (time of season), temperature and margin, and both witnessed the increasing appearance of *G. hederacea*. Rainfall, while had a significant influence on the whole model, had no effect on axes 1 and 2. The presence of precipitation was reflected in species composition to a certain, moderate extent (explained variation <1%) in the open field because *E. crus-galli*, *P. oleracea* and *D. sanguinalis* were found in larger numbers during periods of heavy raining, while many of the common arable weeds including *E. repens* and *Setaria* species appeared in dry intervals. Irrigation, on the other hand, had no significant effect on species composition either in the open-field or in the greenhouse.

**Figure 1:**
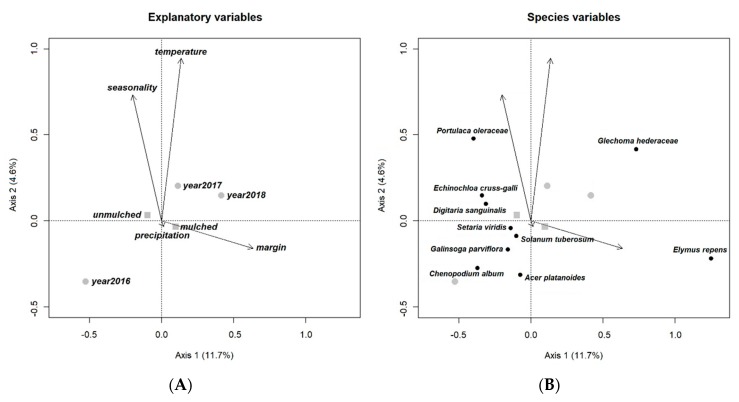
Ordination diagrams of the redundancy analysis (RDA) containing explanatory variables and the weed species to demonstrate connection within explanatory variables (**A**) and between explanatory variables and weed species (**B**) of open field tomato experiment (Gödöllő, Hungary, 2016–2018). Only ten species with the highest weight on the first two RDA axes are presented. Circle = year; square = mulching.

Our ordination diagram reveals that, while margin as a variable had a high influence on the presence of *E. repens* and *G. hederacea*, sampling date (time of season) and temperature influenced *G. hederacea*, *Ch. album*, *E. crus-galli*, and *D. sanguinalis* as well (Figure 1).

### 2.2. Evaluation of Soil Weed Seed Bank

Year had the highest explaining effect with 13% of total explained variation. The explaining effects of other examined variables remained below 2%, but their combined net effect was significant. Among them, mulching resulted in the strongest explained variation (1.85%) (Table 5).

*Ch. album* and *P. oleracea* had the highest germination capacity, while the germination of *S. media* was reduced by mulching. The location of microplots had no influence on the germination of weeds that generally and in our open-field evaluation are more frequent on marginal habitats (*G. hederacea*, *E. repens*, *C. dactylon*). Some annual summer species like *Setaria* sp., *D. sanguinalis* and *S. media*, and the perennial *Taraxacum officinale* Wigg, on the other hand, had lower germination capacities in soil samples taken from microplots closer to the margin. *P. oleracea*, *G. parviflora*, and *D. sanguinalis* germinated with more success from deeper layers, while samples taken from shallow soil layers were more appropriate for the germination of *S. media*, *S. viridis*, and *E. crus-galli* and certain *Amaranthus* species. The highest germination percentage in the first year was observed in the cases of *Ch. album*, *C. arvensis*, *S. pumila*, and *Amaranthus* sp., while the second year had *P. oleracea*, *S. media*, *G. parviflora*, and *T. officinale* germinating in the highest percentage (Table 5).

## 3. Discussion

Mulching alters the micro-environment of the immediate habitat of crops. Mulch materials reduce the availability of light on the soil surface, mulching evens the water content and water availability of the soil by reducing evaporation and enhancing soil structure. Mulching tends to flatten temperature fluctuations as well, as we observed in an earlier, extended study that mulched areas warm up and cool down more gradually when compared to bare soil, air or to non-mulched areas [28].

By altering the environment, mulching and irrigation also influence the prospects of soil biota. As conditions within the soil change, so does the longevity of seeds, the chances of germination of weed seeds, and thus, in turn, the potential of weed seed bank as well [6].

In our experiment, weed mass, or weed mass expressed in the time needed for weeding, was significantly reduced by mulching, and, unlike Law et al. (2006) [29], who found that one mulching at the beginning of the season was not sufficiently able to control weeds throughout the season, we experienced a different tendency: less and less time was needed for weeding as the season progressed. In one of our earlier studies [30], a thick layer of straw provided suitable suppression against weeds, but the thickness of mulch (and year, and time of season) were important factors to determine the efficiency of mulching.

### 3.1. The Impact of Mulching—Standing Weed Composition

It has been shown that mulching not only suppresses weeds, but it also reduces their diversity [22]. Similarly to previous findings, organic mulches of different origin had a different impact on the species composition of perennial weeds; we have also observed the ways mulching transformed weed composition and diversity [25]. Among weed species, the percentage of *Convolvulus arvensis* was rising, and we found two explanations. Since its competitor species were suppressed by mulching, *C. arvensis* had more available space and nutrients for its growth. Secondly, the presence of the mulch layer made weeding difficult. Our findings are similar to one of our earlier studies when straw mulch, even in a thickness of 10 cm was unable to stop the re-growth of *C. arvensis*, and the mulch layer needed to be as thick as 15 cm to have a successful protection [30]. Our findings resemble that of Ozores-Hampton [27] who observed that, in order to obtain a successful weed control by mulching, a minimum thickness of 10–15 centimeters is needed; and with those of Pupalienė et al. [25], but their three-year experiment proved that a 10-cm layer of mulch was efficient in weed control when compared to mulch layers as thin as only 5 cm.

We have to note that the most important means of dissemination for *C. arvensis* is its vines and not its seeds, so our findings match with the following: mulching reduces weeds that multiply by seeds, but cannot control those types of weeds that have vines or other means of multiplication or spread. This is explained by the fact that the presence of a mulching layer inhibits light from entering the surface of the soil [15].

By covering the soil, mulch materials can effectively restrict the emergence of seedlings, but are not as potent against perennial weeds [1]. In their study of the impact of various mulch types and a combination with poly-ethylene cover on the weeds of broccoli and tomato, Kosterna et al. [24] observed that, among annuals, mulching, irrespective of its type, reduced weed mass and the number of weed species. They had *Ch. album*, *E. crus-galli*, and *Veronica arvensis* L. as the most frequent species and had no *C. arvensis* on their list. The most common perennial weed species found was *E. repens* [24], which we recorded only in the third year. Mulching was found to increase species richness, and weed biomass in general was found higher when conventional management practices were compared with a low-input system, where winter cover crops were not ploughed in, but only spread on the surface as mulch [18]. As the authors speculate, this somewhat unique finding may be attributed to the complexity of factors influencing the system and its elements such as weed species composition.

The question of changes in weed species composition, which is, why certain weed species do well despite mulching while others are suppressed, was investigated by Creamer et al. [20], who reflected on the light sensitivity of weed species, because their number is easily reduced regardless of the type of organic mulching material. On the other hand, some other species may take advantage of the elements released into the soil during the decomposition process of mulch residues.

### 3.2. The Impact of Mulching—Weed Seed Bank

Organic mulch, the layer of decomposing material, creates an obstacle between the seed and the surface. Mulching usually means the lack of conventional tillage. As Moonen and Bàrberi [18] pointed out, where there is no tillage, the soil surface becomes a relatively undisturbed habitat not only for predators of agricultural pests known to agriculture, but for insects, small birds, and rodents that predate on weed seeds, causing a direct decrease in the weed seed bank over time. In their more recent study, Moonen & Bàrberi (2006) [19] found that the weed species they studied (*A. retroflexus* and *E. crus-galli*) showed sensitivity not only to the presence of mulch, but to the type of mulch as well.

Our germination studies revealed that *S. media* has lost some of its germination potential, but *Ch. album* and *P. oleracea* remained germinative regardless of the presence of a mulch layer. In this situation, germination of weeds is inhibited by blocking light, as Battla and Benech-Arnold [12] showed about weed species that are positioned near to the surface. In our experiment, *S. media*, *S. viridis*, *E. crus-galli*, and *Amaranthus* sp. germinated from the shallower soil layers, while *P. oleracea*, *G. parviflora*, and *D. sanguinalis* were located deeper within the soil. There is a definite difference in the “soil depth of origin” for the species involved. As Bibbey [31] observed, more seeds germinate from deeper layers of soil than from closer to the surface. Some hold the opposite allegation: germination of certain species is easier on the soil surface than in the soil [12,32].

While organic mulch, regardless of its mass and thickness, had no significant effect on the germination of annual weeds [25], our results showed that both *Setaria* species and *T. officinale* were negatively affected by mulching both in the open field and in the greenhouse. *T. officinale* multiplied by seeds and regrowth on the field, and only by seeds in the greenhouse, meaning mulching hindered both types of multiplication. Interestingly, there was a difference between the level of impact of mulching on the two *Setaria* species. *S. viridis* was better controlled by mulching on the field, while, in the greenhouse, *Setaria pumila* (Poir.) Roem. & Schult. was more severely inhibited when compared to *S. viridis*. One may speculate that the reason behind this can be the differences between environmental demands of these two species: while *S. viridis* seems to prefer the circumstances of closed environments, higher relative humidity values, for example, *S. pumila* seems to prefer open spaces and does not thrive well in the greenhouse.

Since organic mulching means endogenous decomposition processes, the presence of organic mulch layer induces chemical changes too. Moonen and Bàrberi [19] give account of the importance of the release and production of phytochemicals and similar substances, and they relate it to the amount of mulch applied, rather than to the type of mulch. This finding suggests that organic mulches may have a certain amount of inhibition even after they decomposed; and contradicts the studies of Pupalienė et al. [25] who emphasize the significance of the physical limitations mulching represents to weeds. Moonen and Bàrberi [19] expand their idea on the chemical properties of mulching saying that weed seeds need to come into direct contact with the decomposing materials of mulches, but, at the same time, crop seeds need protection.

Among physical properties mulching influences not only the availability of light, but the temperature of the soil, and Kruk et al. [14] citing earlier studies mention that while thermal fluctuations may affect the germination of weeds, its effect is not related to soil depths where weed seeds are located. As we have found as well, mulched plots tend to have smaller ranges of temperature changes [28], and we assume that the impact of mulch is greater on weeds that are sensitive to light, or whose germination is triggered by light, when the seeds are located in the upper layers of the soil.

Weeds that yield larger seeds were less influenced by the environmental changes induced by mulching because seed size is more dependent on species than on environmental factors, or, put in another way: survival time and optimal soil depth for germination is inherently different for a species with larger seeds than for those with smaller seeds.

### 3.3. The Impact of Irrigation and Water Availability—Weed Composition

We found that rainfall only had influence on species composition in the open field, and its influence was only moderate. In a study investigating the connection between light exposure and water availability as expressed in seedling emergence of two weed species by Botto et al. [33], exposure to light encouraged germination only when the plots were irrigated. It suggests that light-sensitive species will take advantage of available water and will not germinate well during dry spells, whereas, in the case of species that do not require water for their germination, water will not be a limiting factor. It also suggests that, when the weed species composition of a field is relatively constant, irrigation, or the lack of it may increase or decrease weed infestation, depending on the light sensitivity of weed species. Since mulch restricts light from reaching any seed that may be present on the surface, we expected weeds to follow the above suggested pattern. Instead, we found that not all weeds that rank high on the Borhidi [34] 1–9 scale of light sensitivity (LB) followed the pattern because, although *E. crus-galli*, *P. oleracea*, and *D. sanguinalis*, which rank high on the LB value with 8, 7 and 7, respectively, took advantage of the high amount of available water and fit into this conception; *E. repens* and *Setaria* species were more frequent in dry periods, although their LB value is between 7 and 8.

### 3.4. The Impact of Irrigation and Water Availability—Weed Seed Bank

We found no strong correlation between the availability of water and the germination of weeds, although increasing moisture content of the soil was observed to favor the germination and early development of weeds [24], when in a study using polypropylene cover, initial weed infestation was higher in covered plots, due to the combination of elevated temperature and water availability within.

### 3.5. The Impact of Margins—Weed Composition

Microplot location had no influence on the presence of weeds that usually prefer undisturbed habitats such as margins (*G. hederacea*) or have a shallow vine system but are frequent in disturbed and undisturbed habitats as well (*E. repens* and *C. dactylon*). The fact that weeds that are more frequent in margins and were generally found in large amounts in our open-field experiment failed to display high levels of germination in pots with soil samples taken from microplots around the margin is not a contradiction, since *G. hederacea*, *E. repens*, and *C. dactylon* prefer to proliferate by means of vegetative propagules that remain within the soil. Oftentimes, the propagules of these weeds are located outside the field and the plants invade the fields during the season.

### 3.6. The Impact of Margins—Weed Seed Bank

We noticed that certain summer annuals such as *Setaria* sp., *D. sanguinalis*, and *S. media* germinated more abundantly when the soil was gathered from the middle of the field. Since these weed species are more frequent in agricultural areas than in margins, they did not penetrate our field from adjacent areas.

### 3.7. The Impact of Time of Season and Succession—Weed Composition and Weed Seed Bank

One of our earlier studies has shown that in all three years, total weed cover was significantly influenced by time of season, or sampling date, and year or succession of years [30]. As the season progressed, the number of species declined, and species composition shifted. At the beginning of the vegetation period *E. repens* was highly abundant, but later, the species almost disappeared. The frequency of *D. sanguinalis* and *G. hederacea* on the other hand, was gradually increasing. Since the sampling period took place between early June and late August, our observations may either relate to the availability of sunlight, that is, the light requirement of these weed species, or to temperature. While *P. oleracea*, *C. arvensis*, and *D. sanguinalis* were found more frequently during warmer weather sections, *S. media* preferred cold periods. The ability of mulching to regulate the temperature of its microhabitat [28] may have contributed to this pattern.

The succession of years also caused a shift in species composition. In fact, year had the highest explained value, accounting for 13% of all the variances. We observed a transition in the flora between 2016 and 2018. In the first year, the most important species were *S. media*, *D. sanguinalis*, *Ch. album*, *G. parviflora*, and *Setaria* sp., which are considered the most frequent weeds of arable fields. Changes started to show with the second year, when the shallow-rooted *S. media* and *G. parviflora* that are frequent in orchards, and *T. officinale* that prefers undisturbed habitats were the most dominant. We also had *G. hederacea* appear in the species list. In the third year, we recorded *E. repens* and *C. dactylon*. We assume that mulching alone and perhaps its combination with irrigation induced a shift in the micro-conditions of the field. The typical arable weeds of the first year reflected the management of previous years, and the impact of mulching on weeds began to show from the second year on into our experiment. We have to mention that conditions have shifted, as from the second year on, experimental microplots had a tomato monoculture cultivation on them.

## 4. Materials and Methods

### 4.1. Open-Field Experiment

Our study microplots were located on the premises of the Plant Protection Institute of Szent István University Gödöllő, Hungary at the Experimental Field (47°35’21,97” N 19°22’03.58” E). The dominant soil type of the Experimental Field is coarse sand. Trials were conducted during the growing seasons of 2016, 2017, and 2018. We had the Hungarian landrace tomato “Dány” (RCAT057829) as crop every year. Previous cultivation in the experimental area between 2011 and 2015 comprised of typical arable crops such as sunflower (*Helianthus annuus* L.), corn (*Zea mays* L.), potato (*Solanum tuberosum* L.), and winter wheat (*Triticum aestivum* L.). Soil management was done by ploughing and rotational tiller, and no herbicides or fertilizers were applied.

In order to study the role of mulch, irrigation, and their combination, there were three treatments: mulch only, irrigation only, mulch and irrigation combined, and the untreated control arranged in a systematic block design so as to avoid that two adjacent microplots receive the same treatment. There were six replications to treatments and the control, resulting a set of 24 (6 × 4) microplots, bordered by a permanent, mown grassland. The location and design of microplots was the same every year in order to detect the potential long-term effect of treatments. Since no microplots received soil work such as ploughing during the three years, the top 10 cm of the treated microplots was considered to display the same soil layer as the control microplots.

In order to separate treatments and provide the exact same size for each microplot, a pinewood frame was constructed in the field to obtain the necessary 24 microplots measuring 2 × 2 m on a total area of 96 m^2^. In every microplot, 4 plants were planted, so every plant had a 1 m^2^ area.

Mulching protocol: Our mulch material was leaf litter of various deciduous tree species, mostly Norway maple (*Acer platanoides* L.), common oak (*Quercus robur* L.), and sycamore (*Platanus orientalis* L. var. *acerifolia* Aiton), which tree species are common in the area. Leaf litter was provided by Zöld Híd B.I.G.G. Non-profit Kft. (Gödöllő, Hungary) in the first year. For mulching in 2017 and 2018, leaves of various tree species dominated by those mentioned above were collected from the inner yard and park of Szent István University (Gödöllő, Hungary). Mulching material was collected in the fall and was stored until its use in the spring in uncovered piles. Mulching material was evenly spread before planting on the soil surface, without being incorporated into the soil, in a thickness of 15 cm.

Irrigation protocol: water was supplied to microplots three times a week by a micro irrigation system consisting of drip irrigation lines and individual drippers at each plant. The amount of supplied water was calculated upon the actual amount of rainfall, following the method of Helyes and Varga [35].

#### 4.1.1. Monitoring Weed Species Composition

Every year (2016, 2017, and 2018) there were five weed surveys during the growing seasons. Microplots were weeded 10 days prior to weed mapping, so each time the growth of 10 days was surveyed. Mapping microplots involved recording weed cover expressed in the percentage of the total area of the microplot (Table 6). After each survey, microplots were hand-weeded or hoed, and the length of weeding time was recorded.

#### 4.1.2. Evaluation of the Weed Seed Bank

Before the growing season, for both 2017 and 2018 to check the effect of open-field mulching of the previous years (2016 and 2017), soil samples were taken from three different depths (0–10 cm, 10–20 cm and 20–30 cm) of every microplot of our open-field tomato experiment and were kept in separate pots per microplot per soil depth in a greenhouse that had natural light, but no precipitation. In order to avoid the desiccation of the soil and help germination, soil samples were irrigated regularly.

Germinated weed seedlings were surveyed five times for samples collected in 2017 and four times for the 2018 samples, because at one point of time in both years, when no more germination occurred, soil samples were disturbed to promote further germination.

In every pot, germinated weed seedlings were counted, and each specimen was identified to species level. Identified monocotyledonous and dicotyledonous seedlings were removed to avoid confusion in the coming further surveys. Perennials, however, were left within because we would not have been able to fully remove their propagules from the pots, and this may have led to false repetitions in further surveys.

Seedlings that were found to have died off at the time of a survey were assigned “unknown species”. Seedlings that were too young for identification at the species level at the time of a survey were either assigned to higher taxa or assigned “waiting” and left growing until being fit for complete identification in a consecutive survey. All weeds assigned “waiting” were identified later during the study.

### 4.2. Data Analyses

Our paper includes two separate analyses: a weed survey of the open-field experiment and a seed bank test (a weed germination survey based on soil samples).

In addition to primary variables of the experiment (mulching and irrigation), we included the following parameters: margin, year, seasonality within year, temperature and precipitation, all with potential influence on germination, as explanatory variables to support the use and versatility of the experimental setting. The model we set up may serve as an indirect tool to prove the impact of mulching on germination. The effect of the bordering vegetation (mown grassland) around the experimental field was included in the model as a ‘margin’ effect, in order to reflect how perennial weeds can invade the marginal microplots.

The model of the open-field survey included the following explanatory variables: mulching (factorial variable; yes or no), irrigation (numeric variable; the total amount of water in mm in three weeks before survey), margin (interval variable; number of sides of microplots directly bordered by the mown grassland margin: 0, 1 or 2), year (factorial variable; 2016, 2017 or 2018), seasonality (interval variable; 1–5, representing the order of weed survey within each year), precipitation (numeric variable; the total amount of rainfall in mm in three weeks before each survey) and temperature (numeric variable; the average of mean daily temperatures in °C of three weeks before each survey).

The model of weed seed bank test included the following explanatory variables: mulching (factorial variable; yes or no), irrigation (factorial variable; yes or no), margin (interval variable; 0, 1, or 2), year (factorial variable; 2017 or 2018).

Both analyses started, with performing a multivariate analysis to determine the average community composition of every field. Then, for every field, we averaged the cover values of weed species across all the four microplots. Cover values were then subjected to a Hellinger transformation [36] and were examined in a redundancy analysis (RDA), together with management and environmental factors. Following the procedure described by Legendre and Gallagher [36], this relates species data to explanatory variables more accurately than the canonical correspondence analysis (CCA), even if the species response curves are unimodal (owing to, e.g., long gradients). The number of explanatory variables was reduced by stepwise backward selection using a *p* < 0.05 threshold for type I error, which led to a minimal adequate model containing 6 terms (in the case of open-field experiment) and 4 terms (in the case of soil seed bank test). The sole excluded variable was the irrigation in both open-field and soil seed bank test.

As a next step of the multivariate analysis, we assessed gross and net effects of each explanatory variable of the reduced model, according to the methodology of Lososová et al. [37]. The gross effect of a variable was defined as the variation explained by a ‘univariate’ RDA containing the studied predictor as the only explanatory variable. The net effect, on the other hand, was assessed as the significance of a similar partial RDA (pRDA) with the studied predictor still being the only constraining variable, but all the other variables of the reduced model were also involved as conditioning variables (‘co-variables’), the effect of which was ‘partialled out’ (i.e., removed before the actual RDA). In the case of net effects, model significances were assessed as type I error rates were obtained by permutation tests. There was only one constrained axis in the partial RDAs, except for analyzing year in the case of open-field test, where there were two constrained axes (number of categories—1), and all axes were tested separately [38].

Based on these results, a common rank of ‘importance’ was established among all explanatory variables according to the R2 adj-values of the net effects of the pRDA models. To demonstrate the responses of the weed species to the individual significant management and environmental factors, in each case, we identified 10 species (with >3 occurrences) that expressed the highest explained variation by the constrained axis in the partial RDA.

To check for the presence of spatial autocorrelation or spatially structured species–environment relationship, we applied spatial partitioning of ordination results [39]. Having found no significant spatial effects, we followed the analysis without considering spatial position of fields. Intercorrelations of model terms were checked prior to the analysis by calculating variance inflation factors [40]. Significant variables showed only slight intercorrelations, which do not bias the analysis, the highest GVIF (generalized variance inflation factor) score adjusted by a degree of freedom was 2.51 (in the case of an open-field experiment) and 1.01 (in the case of a soil seed bank test).

In the RDA ordination diagrams of the reduced model, co-ordinates of continuous variables were calculated from their linear constraints, while categorical variables were transformed to ‘dummy’ indicator variables, and these dummies were placed in the ordination space by weighted average calculations.

Both numeric and factorial variables were tested by Analysis of Covariance (ANCOVA) in the case of total weed coverage and weeding time during open-field experiments. In significant cases, explanatory variables were tested by a two-samples *T*-test, or by a Tukey comparison for factorial variables and by Pearson correlation for numeric variables.

The entire statistical analysis was performed in the R Environment (R Development Core Team, version 3.5.3) using the Vegan and Car add-on packages (vegan 2.5-4 and car 3.0-2).

## 5. Conclusions

Weed composition and weed seed bank of a field are influenced by a combination of a multitude of environmental and management factors. As Menalled [7] noted, integrated weed management needs the support of the knowledge of these factors and their interactions.

Among the factors we investigated, mulching had the most pronounced impact on weeds. The impact of margins was significant, but impact of the availability of water, whether coming from natural precipitation or from irrigation, was not.

Our experience shows that covering the soil surface with the leaf litter of deciduous trees modifies the survival chances of weeds and is able to reduce both the coverage and the diversity of weeds and mulching time required, and these may all have a great impact on the success of crop production.

A cropping system directly affects the germination, persistence, and mortality of weeds [6], and as mulching can be an element of a cropping system with or without tillage, mulching is a tool to manage weeds and their seed bank in an environmentally acceptable agricultural system.

Zaniewicz-Bajnowska et al. [23] found a correlation between the suppressive effect of mulch on the volume of weeds, and the type of crop protected from weed infestation. Based on their findings, we suggest further studies to fine-tune mulching protocols to match the specific needs of targeted crops.

## Figures and Tables

**Table 1:** Gross and net effects of explanatory variables on weed composition in an open-field tomato experiment using redundancy analysis (RDA) with single explanatory variables (Gödöllő, Hungary, 2016–2018).

	Gross Effect			Net Effect			
Factors	d.f.	Explained Variation (%)	R^2^_adj_	Explained Variation (%)	R^2^_adj_	F	*p*-Value
Mulching	1	3.669	0.03400	3.681	0.03533	16.939	0.001
Margin	1	5.813	0.05550	5.835	0.05729	26.849	0.001
Seasonality	4	3.128	0.02857	1.004	0.00802	4.620	0.001
Year	2	8.922	0.08412	8.300	0.07999	19.097	0.001
Rainfall	1	0.661	0.00384	0.855	0.00650	3.932	0.001
Temperature	1	3.097	0.02827	0.722	0.00515	3.321	0.002
Irrigation	1	0.807	0.00530	0.481	0.00269	2.212	0.019

**Table 2:** Names, score values and fit of species giving the highest fit along the first constrained axis in the partial redundancy analysis (pRDA) models in an open-field tomato experiment (Gödöllő, Hungary, 2016–2018).

	Ax 1 Score	Fit		Ax 1 Score	Fit
**Mulching (+ yes; − no)**			**Rainfall (+ high; − low)**		
*Convolvulus arvensis*	0.3296	0.0749	*Echinochloa crus-galli*	0.1486	0.0253
*Conyza canadensis*	−0.0633	0.0370	*Portulaca oleracea*	0.1167	0.0132
*Setaria viridis*	−0.0710	0.0271	*Digitaria sanguinalis*	0.0942	0.0170
*Galinsoga parviflora*	−0.1102	0.0453	*Sonchus asper*	0.0092	0.0105
*Echinochloa crus-galli*	−0.1282	0.0188	*Solidago canadensis*	−0.0163	0.0322
*Digitaria sanguinalis*	−0.1295	0.0322	*Galinsoga parviflora*	−0.0436	0.0071
*Chenopodium album*	−0.1587	0.0290	*Setaria pumila*	−0.0589	0.0235
*Glechoma hederacea*	−0.1669	0.0164	*Solanum tuberosum*	−0.0651	0.0323
*Taraxacum officinale*	−0.2355	0.0590	*Setaria viridis*	−0.0682	0.0250
*Portulaca oleracea*	−0.3994	0.1553	*Elymus repens*	−0.1477	0.0105
**Weeding date (+ late; − early)**			**2016 (+ high; − low)**		
*Elymus repens*	−0.2319	0.0259	*Stellaria media*	0.235	0.050202
*Solanum tuberosum*	−0.0665	0.0337	*Digitaria sanguinalis*	0.228	0.100282
*Acer platanoides*	−0.0397	0.0067	*Chenopodium album*	0.227	0.059332
*Galinsoga parviflora*	−0.0381	0.0054	*Galinsoga parviflora*	0.161	0.096344
*Conyza canadensis*	−0.0316	0.0092	*Setaria viridis*	0.130	0.091409
*Amaranthus retroflexus*	−0.0204	0.0079	*Acer platanoides*	0.118	0.058729
*Solidago canadensis*	−0.0137	0.0228	*Setaria pumila*	0.097	0.064132
*Polygonum aviculare*	−0.0128	0.0072	*Solanum tuberosum*	0.095	0.069209
*Digitaria sanguinalis*	0.0617	0.0073	*Elymus repens*	−0.495	0.118287
*Glechoma hederacea*	0.1417	0.0118	*Glechoma hederacea*	−0.517	0.156645
**Temperature (+ high; − low)**			**2017 (+ high; − low)**		
*Portulaca oleracea*	0.2062	0.0414	*Glechoma hederacea*	0.2879	0.0487
*Convolvulus arvensis*	0.1303	0.0117	*Echinochloa crus-galli*	0.2759	0.0872
*Digitaria sanguinalis*	0.0949	0.0173	*Conyza canadensis*	0.0508	0.0238
*Solanum tuberosum*	0.0334	0.0085	*Trifolium repens*	0.0255	0.0103
*Solidago canadensis*	0.0104	0.0130	*Polygonum aviculare*	−0.0164	0.0119
*Medicago lupulina*	−0.0097	0.0063	*Solanum tuberosum*	−0.0477	0.0173
*Chelidonium majus*	−0.0159	0.0125	*Cynodon dactylon*	−0.0523	0.0137
*Conyza canadensis*	−0.0425	0.0167	*Setaria viridis*	−0.0750	0.0302
*Acer platanoides*	−0.1048	0.0465	*Digitaria sanguinalis*	−0.0998	0.0191
*Stellaria media*	−0.2645	0.0637	*Stellaria media*	−0.2394	0.0522
**Margin (+ marginal area; − central area)**			**2018 (+ high; − low)**		
*Elymus repens*	0.6448	0.2004	*Elymus repens*	0.4424	0.0943
*Glechoma hederacea*	0.3932	0.0907	*Glechoma hederacea*	0.2287	0.0307
*Cynodon dactylon*	0.0310	0.0048	*Cynodon dactylon*	0.1045	0.0549
*Medicago lupulina*	0.0124	0.0102	*Amaranthus retroflexus*	−0.0311	0.0183
*Sonchus asper*	0.0097	0.0115	*Setaria pumila*	−0.0652	0.0288
*Solanum tuberosum*	−0.0458	0.0160	*Acer platanoides*	−0.0865	0.0317
*Taraxacum officinale*	−0.0909	0.0088	*Digitaria sanguinalis*	−0.1287	0.0318
*Chenopodium album*	−0.0932	0.0100	*Galinsoga parviflora*	−0.1294	0.0625
*Portulaca oleracea*	−0.1836	0.0328	*Chenopodium album*	−0.3077	0.1090
*Convolvulus arvensis*	−0.2864	0.0566	*Echinochloa crus-galli*	−0.3826	0.1678

**Table 3:** Effect of explanatory variables on weeding time (min/microplot) in an open-field tomato experiment (Gödöllő, Hungary, 2016–2018).

**Factor Variable**	**d.f.**	**ANCOVA**		**Tukey Comparison**		
		**F**	***p*-Value**	**Group**	**Avg Value (min/microplot)**	**Sign. Class**
Mulching	1	79.814	0.000	mulched	5.41	a
				unmulched	9.63	b
Year	2	82.743	0.000	2016	11.08	c
				2017	7.81	b
				2018	3.67	a
**Numeric/Interval Variable**	**d.f.**	**ANCOVA**		**Pearson Correlation**		
		**F**	***p*-Value**	**Corr.**		***p***
Weeding date	1	22.335	0.000	−0.197		0.001
Rainfall	1	25.969	0.000	−0.335		0.000
Margin	1	0.061	ns	-		-
Temperature	1	3.518	ns	-		-
Irrigation	1	0.607	ns	-		-

**Table 4:** Effect of explanatory variables on total weed coverage (%) in an open-field tomato experiment (Gödöllő, Hungary, 2016–2018).

**Factoral Variable**	**d.f.**	**ANCOVA**		**Tukey Comparison**		
		**F**	***p*-Value**	**Group**	**Avg Value (%)**	**Sign. Class**
Mulching	1	8.21	0.004	mulched	7.35	a
				unmulched	11.59	b
Year	2	20.034	0.000	2016	13.32	b
				2017	11.40	b
				2018	3.69	a
**Numeric/Interval variable**	**d.f.**	**ANCOVA**		**Pearson Correlation**		
		**F**	***p*-Value**	**Corr.**		***p***
Sampling date	1	66.345	0.000	−0.405		0.000
Rainfall	1	0.103	ns	-		-
Margin	1	0.07	ns	-		-
Temperature	1	9.874	0.00186	−0.486		0.000
Irrigation	1	1.32	ns	-		-

**Table 5:** Gross and net effects of explanatory variables on weed seed bank in an open-field tomato experiment identified using redundancy analysis (RDA) with single explanatory variables (Gödöllő, Hungary, 2016–2018).

	Gross Effect			Net Effect			
Factors	d.f.	Explained Variation (%)	*R* ^2^_adj_	Explained Variation (%)	*R* ^2^_adj_	*F*	*p*-Value
Mulching	1	1.820	0.01119	1.848	0.01272	3.067	0.003
Margin	1	1.172	0.00466	1.094	0.00502	1.815	0.044
Depth	4	1.491	0.00787	1.502	0.00919	2.494	0.006
Year	1	12.994	0.12372	13.012	0.12679	21.598	0.001

**Table 6:** Timeline of actions during study years (open-field tomato, Gödöllő, Hungary, 2016–2018).

		Year	
	2016	2017	2018
Planting	2 June	12 May	9 May
Mulching	18 March	17 March	9 May
Harvest	30 August	19 September	26 September
Rainfall (during the growing season)	213 mm	299.5 mm	370.5 mm
Irrigation water	153 mm	303.2 mm	193.4 mm
Average temperature	21.0 °C	21.1 °C	21.6 °C
Minimum temperature	8.6 °C	7.0 °C	0.0 °C
Maximum temperature	35.0 °C	38.0 °C	35.0 °C
Weed survey and Weeding	26 May	2 June	5 June
	27 June	23 June	26 June
	18 July	18 July	18 July
	5 August	6 August	9 August
	28 August	26 August	4 September
